# Innate Immunity Drives the Initiation of a Murine Model of Primary Biliary Cirrhosis

**DOI:** 10.1371/journal.pone.0121320

**Published:** 2015-03-25

**Authors:** Chao-Hsuan Chang, Ying-Chun Chen, Weici Zhang, Patrick S. C. Leung, M. Eric Gershwin, Ya-Hui Chuang

**Affiliations:** 1 Department of Clinical Laboratory Sciences and Medical Biotechnology, College of Medicine, National Taiwan University, Taipei, Taiwan; 2 Rheumatology, Allergy and Clinical Immunology, University of California at Davis, Davis, CA, 95616, United States of America; University of Cincinnati College of Medicine, UNITED STATES

## Abstract

Invariant natural killer T (iNKT) cells play complex roles in bridging innate and adaptive immunity by engaging with glycolipid antigens presented by CD1d. Our earlier work suggested that iNKT cells were involved in the initiation of the original loss of tolerance in primary biliary cirrhosis (PBC). To address this issue in more detail and, in particular, to focus on whether iNKT cells activated by a Th2-biasing agonist (2s,3s,4r)-1-*O*-(α-_D_-galactopyranosyl)-*N*-tetracosanoyl-2-amino-1,3,4-nonanetriol (OCH), can influence the development of PBC in a xenobiotic-induced PBC murine model. Groups of mice were treated with either OCH or, as a control, α-galactosylceramide (α-GalCer) and thence serially followed for cytokine production, markers of T cell activation, liver histopathology and anti-mitochondrial antibody responses. Further, additional groups of CD1d deleted mice were similarly studied. Our data indicate that administration of OCH has a dramatic influence with exacerbation of portal inflammation and hepatic fibrosis similar to mice treated with α-GalCer. Further, iNKT cell deficient CD1d knockout mice have decreased inflammatory portal cell infiltrates and reduced anti-mitochondrial antibody responses. We submit that activation of iNKT cells can occur via overlapping and/or promiscuous pathways and highlight the critical role of innate immunity in the natural history of autoimmune cholangitis. These data have implications for humans with PBC and emphasize that therapeutic strategies must focus not only on suppressing adaptive responses, but also innate immunity.

## Introduction

Invariant natural killer T (iNKT) cells, expressing semi-invariant Vα14-Jα28 chain preferentially pair with Vβ2, Vβ7, or Vβ8.2 chain, hypersecrete Th1 and Th2 cytokines and chemokines upon stimulation with an appropriate ligand, such as α-galactosylceramide (α-GalCer) [1,2]. Activation of iNKT cells *in vivo* leads to subsequent activation of other cells, such as dendritic cells, NK cells, T cells and B cells. Through the release of specific types of cytokines, iNKT cells regulate a cascade of immune reactions that alter the balance of subsequent Th1 and Th2 responses [3]. α-GalCer is a well-defined potent and specific ligand for iNKT cell activation in both humans and mice. Upon ligation of their invariant T cell receptors with α-GalCer presented by CD1d of antigen presenting cells, iNKT cells rapidly produce large amount of cytokines, including IFN-γ and IL-4 [4,5,6].

Moreover, modification of the length of the lipid chain of α-GalCer results in the generation of glycolipids with predominant Th1 or Th2 cytokine skewing profiles [7]. (2s,3s,4r)-1-*O*-(α-_D_-galactopyranosyl)-*N*-tetracosanoyl-2-amino-1,3,4-nonanetriol (OCH), a synthetic analog of α-GalCer with a truncated sphingosine chain, stimulates iNKT cells to produce predominantly Th2 cytokines [8]. Studies using models of experimental autoimmune diseases, including arthritis, diabetes, and experimental autoimmune encephalomyelitis (EAE), have indicated that activation of iNKT cells by OCH ameliorates or prevents these Th1-mediated diseases, which was attributed to its induction of IL-4 and Th2 skewing [9,10,11,12].

Primary biliary cirrhosis (PBC) is a progressive autoimmune liver disease with female predilection characterized by immune-mediated destruction of intrahepatic small bile ducts, leading to decreased bile secretion, fibrosis, and eventual liver failure [13,14,15]. Although the role of cytokines in the pathogenesis of PBC remains unresolved [16,17], PBC is considered a Th1 and/or Th17 dominant autoimmune responses similar to other organ-specific autoimmune diseases [13,14,15] and activation of natural killer T cells accelerates PBC in murine models [18,19,20]. Further, iNKT cell activation by α-GalCer leads to a profound exacerbation of portal inflammation, granuloma formation, bile duct damage, and hepatic fibrosis in a chemical xenobiotic murine model of PBC [18,19]. Despite major advances in our understanding of the immunobiology of PBC in both humans and murine models, there remain major voids in our dissection of initiating and exacerbating factors [21,22,23,24,25,26,27].

In this study, we investigated the effect of administration of OCH on the natural history of xenobiotic induced autoimmune cholangitis. We report herein that mice administered with OCH have exacerbated portal inflammation and hepatic fibrosis similar to mice treated with α-GalCer. iNKT cell deficient CD1d knockout mice immunized with 2-OA-BSA have reduced anti-mitochondrial antibody (AMA) production and cell infiltrates in the liver compared to that of immunized wild type mice. Thus, activation of iNKT cells is critical for the pathogenesis of autoimmune cholangitis and highlights the important roles of innate immunity in this murine model of PBC.

## Materials and Methods

### Experimental mice

Female C57BL/6 mice aged 8–10 weeks were obtained from the National Laboratory Animal Center, Taiwan. CD1d^-/-^ mice on a B6 background were kindly provided by Dr. Chyung-Ru Wang (Northwestern University Feinberg School of Medicine, Chicago, IL, USA). All mice were maintained in the Animal Center of the College of Medicine, National Taiwan University and all experiments performed following approval of The Institutional Animal Care and Use Committee (IACUC) of National Taiwan University College of Medicine and College of Public Health.

### Experimental protocol

Mice were intraperitoneally immunized with 2-OA-BSA (150μg) in the presence of complete Freund's adjuvant (CFA, Sigma-Aldrich, St. Louis, MO, USA) and subsequently boosted at weeks 2, 4, 6 and 8 with 2-OA-BSA and incomplete Freund's adjuvant (IFA, Sigma-Aldrich). Sera were obtained on all mice at 12 weeks post-immunization and titers of IgM and IgG anti-PDC-E2 autoantibodies measured by ELISA. Mice were sacrificed by carbon dioxide overdose at 12 weeks post immunization for liver histopathology, including mononuclear cell phenotypes. For the glycolipid study, mice were injected intravenously using the retro-orbital route with 2 μg of α-GalCer (KRN7000, Funakoshi, Tokyo, Japan), (2s,3s,4r)-1-*O*-(α-_D_-galactopyranosyl)-*N*-tetracosanoyl-2-amino-1,3,4-nonanetriol (OCH) (Enzo Life Sciences, Farmingdale, NY, USA) or PBS simultaneously with first and second 2-OA-BSA immunizations.

### Determination of anti-PDC-E2 antibodies

Serum and supernatant titers of IgM and IgG anti-PDC-E2 autoantibodies were measured by ELISA using recombinant murine PDC-E2. Briefly, purified recombinant PDC-E2 at 1 μg/ml in carbonate buffer (pH 9.6) was coated onto ELISA plates at 4°C overnight. After blocking with 1% casein for 1 hour, supernatants or diluted sera were added for 2 hours at room temperature. The ELISA plates were washed with PBS-tween 20 followed by the addition of horseradish peroxidase (HRP)-conjugated goat anti-mouse IgG (1:5000, Zymed Laboratories, Carlsbad, CA, USA) and IgM (1:5000, Invitrogen, Camarillo, CA, USA). The plates were incubated for another 1 hr and immunoreactivity detected by measuring optical density (O.D.) at 450 nm after exposure for 20 minutes to tetramethylbenzidine (TMB) substrate (R&D systems, Minneapolis, MN, USA).

### Mononuclear cell preparation

Livers were perfused with PBS containing 0.2% BSA (PBS/0.2% BSA), passed through a 100μm nylon mesh, and re-suspended in PBS/0.2% BSA. The parenchymal cells were removed as pellets after centrifugation at 50 g for 5 minute and the non-parenchymal cells were then isolated using Histopaque-1077 (Sigma-Aldrich). After centrifugation, collected cells were washed with PBS/0.2% BSA and viability of cells was confirmed by trypan blue dye exclusion.

### Flow cytometry

Subsets of liver mononuclear cells were measured by flow cytometry. Before staining with a previously defined optimal dilution of monoclonal antibodies (Abs), the cells were pre-incubated with anti-CD16/32 (clone 93) to block non-specific FcRγ binding. The following Abs were used in this study: anti-CD69, anti-CD44, anti-CD3, anti-CD4, anti-CD8a, anti-CD19, (Biolegend, San Diego, CA, USA), anti-NK1.1 (eBioscience, San Diego, CA, USA) and PE-conjugated mouse CD1d tetramers loaded with PBS57 (PBS57 loaded CD1d tetramer, originally produced by the NIH tetramer facility, and supplied through Dr. David Serreze). Stained cells were assessed on a FACSCalibur (BD Biosciences, San Diego, CA, USA) using FlowJo software (Tree Star, Inc., Ashland, OR, USA). Optimal concentrations of the mAbs were used throughout and all assays included positive and negative controls.

### Histopathology

Portions of liver were excised and immediately fixed with 10% buffered formalin solution for 2 days at room temperature. Paraffin-embedded tissue sections were then cut into 4-μm slices for routine hematoxylin and eosin (H-E) and Masson’s Trichrome staining. Liver inflammation was evaluated using light microscopy using our previously defined scoring system [17,28,29].

### Cytokine expression by liver mononuclear cells

Liver mononuclear cells (2x10^6^/ml) were cultured with 1μg/ml anti-CD3 Ab and 1μg/ml anti-CD28 Ab (Biolegend) for 48 hours. ELISA (Duoset, R&D, Minneapolis, MN, USA) assay applied for detection of IFN-γ production in the culture supernatant.

### Statistical analysis

Data are presented as the mean ±standard error of the mean (SEM). All graphing and statistical analyses were performed using the Prism graphing program (GraphPad Software, San Diego, CA, USA). p-values were calculated using a two-tailed unpaired Mann–Whitney U test. Significance levels were set at a p-value of 0.05.

## Results

### Th2-biased cytokine profile was induced by OCH

**Fig 1:**
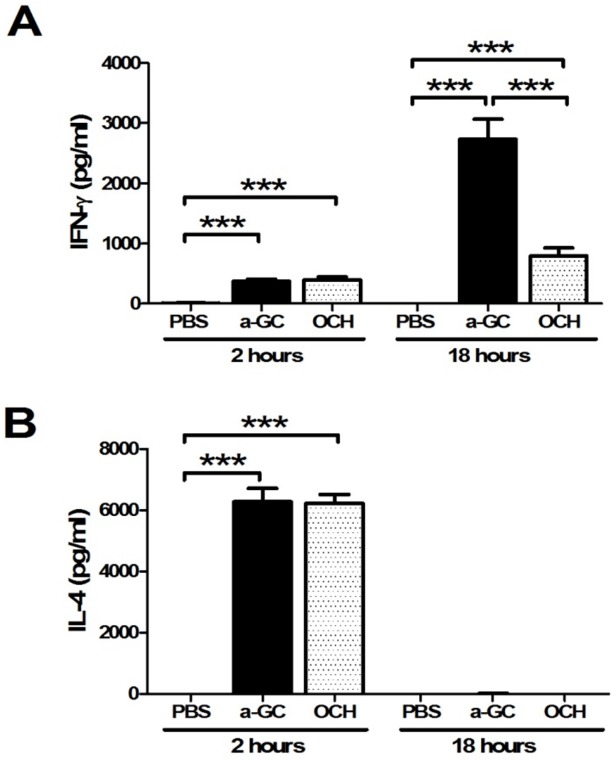
Lower serum levels of IFN-γ after OCH injection. C57BL/6 mice were intravenously injected with α-GalCer, OCH, or PBS. Serum samples were collected at 2 and 18 hours after α-GalCer, OCH, or PBS injection. IFN-*γ* (A) and IL-4 (B) were measured by ELISA. n = 10 mice per group. ***, p<0.001.

As expected and as controls when these studies were initiated, intravenous α-GalCer induced high levels of IL-4 and IFN-γ within 18 hours of injection; neither IFN-γ nor IL-4 were detected in control injected mice (Fig. 1). In OCH injected mice, both IFN-γ and IL-4 were significantly induced and elevated IFN-γ production (391.3 ±165.7 pg/ml) was found at 2 hours. However, significantly lower IFN-γ was found at 18 hours by OCH compared to α-GalCer (794.1±428.4 pg/ml vs. 2735.0 ±1042.0 pg/ml, respectively) (Fig. 1A). A predominant production of IL-4 was detected at 2 hours of OCH administration and it became undetectable at 18 hours (Fig. 1B). IL-17 was undetectable in PBS, α-GalCer or OCH injected mice (data not shown). Thus, these data indicate that α-GalCer initiated a rapid IL-4 production and a prolonged high level of IFN- γ, whereas OCH induced a predominant IL-4 production and a weak IFN- γ level.

### OCH promoted antigen-specific B cell response in 2-OA-BSA immunized mice

**Fig 2:**
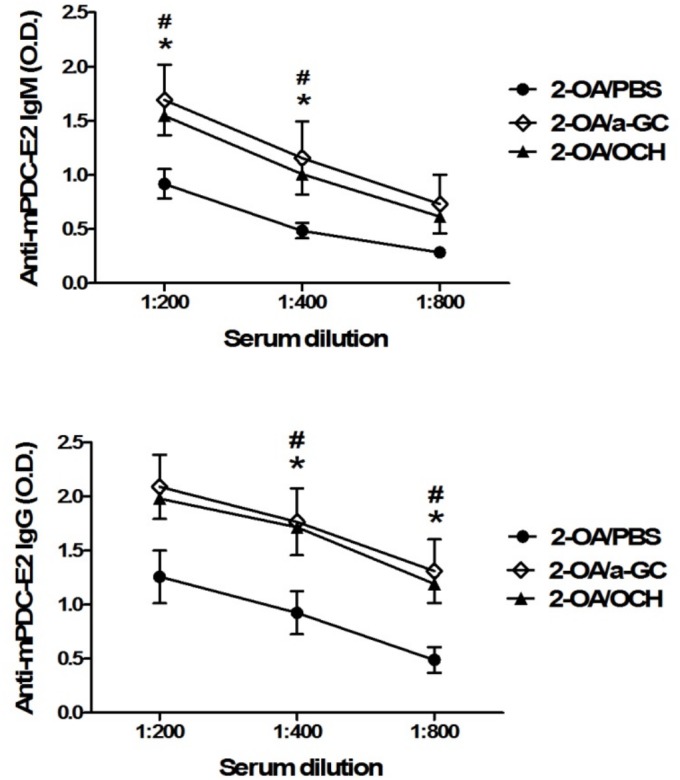
Increased serum AMAs in mice injected with 2-OA-BSA/OCH. Wild type mice were immunized with 2-OA-BSA and α-GalCer (group name: 2-OA/a-GC), OCH (group name: 2-OA/OCH) or PBS (group name: 2-OA/PBS) at weeks 0, 2, 4, 6 and 8. At week 12, serum levels of autoantibodies to mPDC-E2 were measured by ELISA. n = 9–10 mice per group. *, p < 0.05 in 2-OA/a-GC to 2-OA/PBS; #, p < 0.05 in 2-OA/OCH to 2-OA/PBS.

Since α-GalCer and OCH initiate different cytokine profiles, we sought to address whether these two glycolipids induced different antigen-specific B cell responses. As shown in Fig. 2, serum IgM and IgG antibodies to PDC-E2 were increased in 2-OA-BSA/ OCH (2-OA/OCH) immunized mice compared to 2-OA-BSA/PBS (2-OA/PBS) immunized mice. There were no significant differences in the titers of anti-PDCE2 antibodies between 2-OA/a-GC group and 2-OA/OCH group (Fig. 2).

### Increased mononuclear inflammatory infiltrate in OCH injected 2-OA-BSA immunized mice

**Fig 3:**
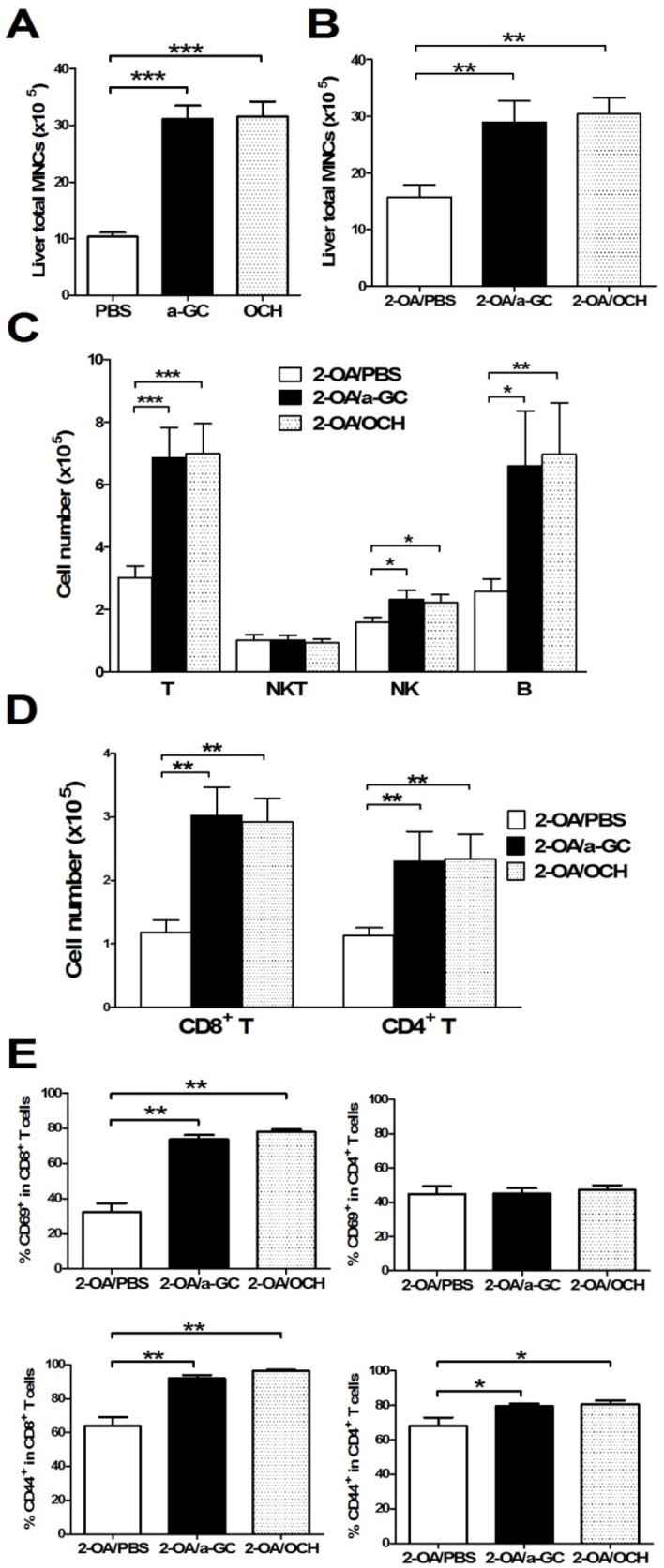
OCH administration increased cell infiltrates and activation of T cells in mice. (A) C57BL/6 mice were intravenously injected with α-GalCer, OCH, or PBS. Liver total mononuclear cells (MNC) were counted 3 days after α-GalCer, OCH, or PBS injection. n = 10–13 mice per group. ***, p<0.001. (B-E) Wild type mice were immunized with 2-OA-BSA and α-GalCer (group name: 2-OA/a-GC), OCH (group name:2-OA/OCH) or PBS (group name: 2-OA/PBS) at weeks 0, 2, 4, 6 and 8 and sacrificed at week 12. (B) Liver total mononuclear cells (MNC) were measured. (C) The numbers of T (CD3^+^ NK1.1^-^), NKT (CD3^+^NK1.1^+^), NK (CD3^-^NK1.1^+^) and B (CD19^+^) cells were measured. (D) The numbers of CD4^+^ and CD8^+^ T cells were detected. (E) The expression of CD69 and CD44 in CD4^+^ and CD8^+^ T cells was measured by flowcytometry. n = 9–10 mice per group. *, p<0.05; **, p<0.01; ***, p<0.001.

**Fig 4:**
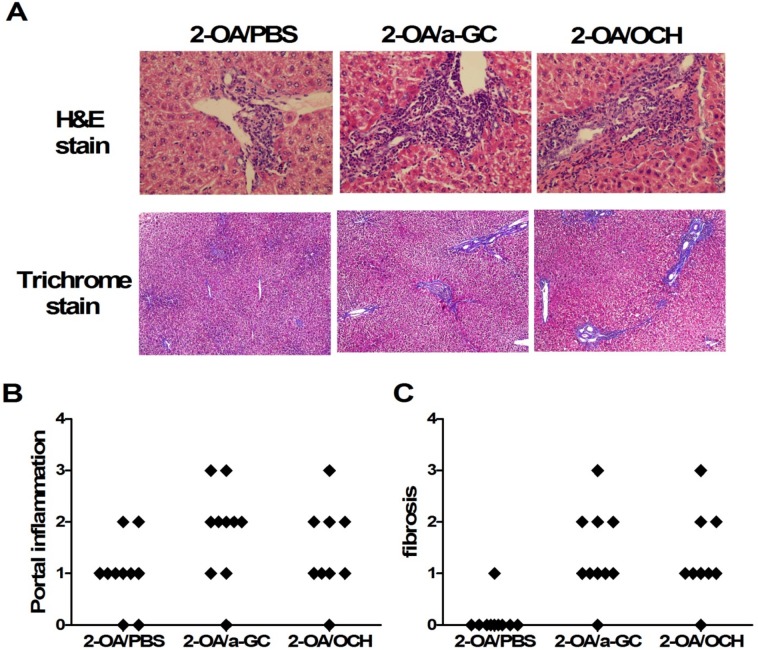
The increase of portal inflammation and fibrosis in mice injected with 2-OA-BSA/OCH. Mice were immunized with 2-OA-BSA and α-GalCer (group name: 2-OA/a-GC), OCH (group name: 2-OA/OCH) or PBS (group name: 2-OA/PBS) at weeks 0, 2, 4, 6 and 8 and sacrificed at week 12. (A) Representative stained liver sections of haematoxylin and eosin (H&E) and Masson’s trichrome stain. (B) Histopathological scores of individual livers on portal inflammation and fibrosis. 0 = no significant change, 1 = minimal, 2 = mild, 3 = moderate, and 4 = severe pathology. Individual symbols each represent a single mouse.

Increased numbers of liver mononuclear cells were noted as early as 3 days after administration with OCH or α-GalCer compared to PBS controls (Fig. 3A), indicating that both glycolipids drive the recruitment of mononuclear cells into the liver. At 12 weeks post immunization, 2-OA-BSA/ OCH immunized mice had significantly higher liver total mononuclear cell infiltrates, increased numbers of T, B and NK cells, and increased CD4^+^ and CD8^+^ T cells compared to 2-OA-BSA/PBS immunized mice (Fig. 3B, C, and D). The frequencies of CD44 expressing CD8^+^ T cells and CD69 expressing CD8^+^ T cells were significantly increased in 2-OA-BSA/OCH immunized mice compared to 2-OA-BSA/PBS immunized mice. Moreover, the frequency of CD44 expressing CD4^+^ T cells was significantly increased in 2-OA-BSA/OCH immunized mice (Fig. 3E). There were no differences in mononuclear cells, cell subsets, and activating T cells between 2-OA/α-GC group and 2-OA/OCH group (Fig. 3). Histologically, there were significant increases in portal inflammation and fibrosis in the 2-OA/OCH group compared to 2-OA/PBS group mice and no differences between the 2-OA/a-GC group and 2-OA/OCH group (Fig. 4). Taken together, not only α-GalCer but, importantly, OCH administration induced more inflammatory cells to liver, including activating CD4^+^ and CD8^+^ T, NK, and B cells, which led to portal inflammation and liver fibrosis.

### Decreased AMAs, cell infiltrates, and IFN-γ production of liver mononuclear cells in 2-OA-BSA immunized CD1d^-/-^ mice

**Fig 5:**
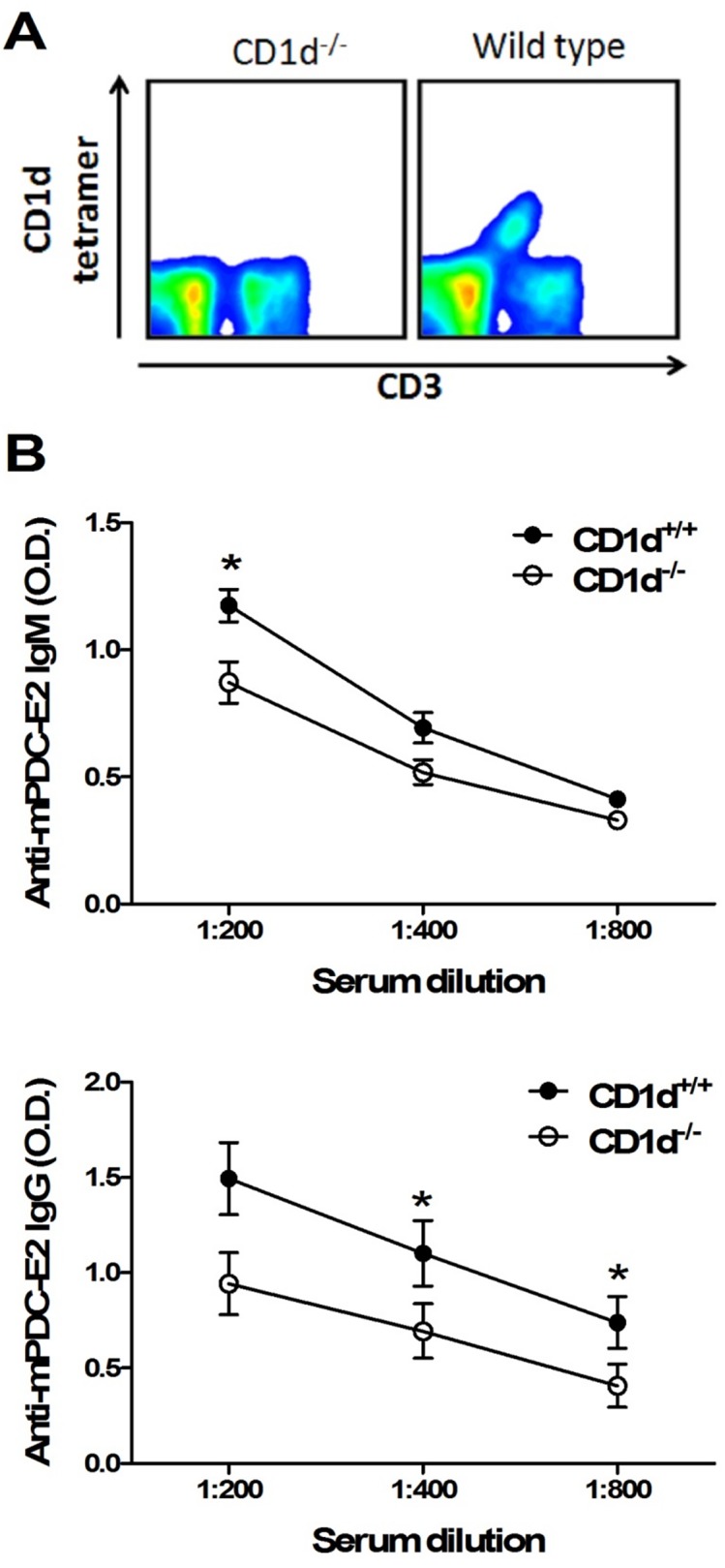
Decreased AMAs in 2-OA-BSA immunized CD1d^-/-^ mice. (A) iNKT cell deficiency in CD1d^-/-^ mice was confirmed by staining with CD3 and CD1d tetramer. (B) CD1d^-/-^ and wild type mice were immunized with 2-OA-BSA at 0, 2, 4, 6 and 8 and sacrificed at week 12. Serum levels of IgM and IgG to mPDC-E2 were measured by ELISA. n = 10 mice for each group. *, p<0.05.

**Fig 6:**
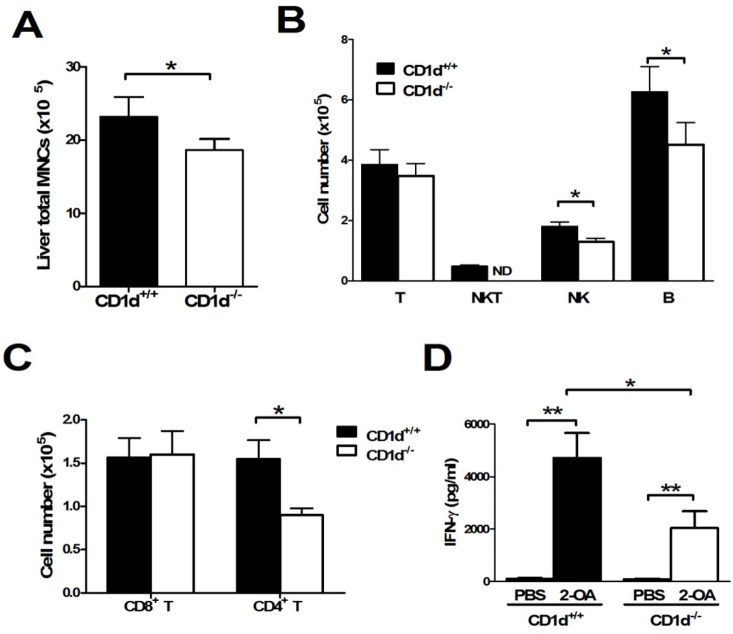
Decreased cell infiltrates and IFN-γ production of liver mononuclear cells in 2-OA-BSA immunized CD1d^-/-^ mice. (A-C) CD1d^-/-^ and wild type mice were immunized with 2-OA-BSA at 0, 2, 4, 6 and 8 and sacrificed at week 12. (A) Liver total mononuclear cells (MNCs) were measured. (B) The numbers of T (CD3^+^ NK1.1^-^), NKT (CD3^+^NK1.1^+^), NK (CD3^-^NK1.1^+^) and B (CD19^+^) cells were measured. (C) The numbers of CD4^+^ and CD8^+^ T cells were detected. n = 16–20 per group. *, p<0.05. (D) CD1d^-/-^ or wild type mice were immunized with 2-OA-BSA at weeks 0, 2, and 4 and sacrificed 3 days after last immunization. IFN-γ production of liver mononuclear cells stimulated with anti-CD3 and anti-CD28 Abs for 2 days was measured by ELISA. n = 8 mice for PBS treated group, n = 13–17 mice for 2-OA-BSA immunized group. *, p<0.05; **, p<0.01.

To further define the role of natural iNKT cells in the pathogenesis of autoimmune cholangitis, we immunized 2-OA-BSA to iNKT cell deficient CD1d knockout mice and monitored disease progress. At 12 weeks, serum IgM and IgG antibodies to PDC-E2 were significantly decreased in 2-OA-BSA immunized CD1d^-/-^ mice as compared to that of wild type mice (Fig. 5B). Liver total mononuclear cell infiltrates were significantly lower in 2-OA-BSA immunized CD1d^-/-^ mice than wild type mice (Fig. 6A). The total numbers of CD4^+^ T cells, NK cells and B cells were significantly decreased in 2-OA-BSA immunized CD1d^-/-^ mice (Fig. 6B and C). Moreover, decreased expression of IFN-γ in activated liver mononuclear cells of 2-OA-BSA immunized CD1d^-/-^ mice was noted compared to wild type mice (Fig. 6D).

## Discussion

Cytokine secretion profile, timing of activation, and type of lipid antigens are important factors determining the pathogenic or protective function of NKT subsets after *in vivo* stimulation and activation [3,30,31]. In xenobiotic immunized mice, iNKT cell activation by a synthetic glycoplipid, such as α-GalCer, leads to the exacerbation of portal inflammation, granuloma formation, bile duct damage and in particular hepatic fibrosis [18,19]. Moreover, *N*. *aromaticivorans* is a microorganism that expresses the conserved mammalian PDC-E2 autoepitopes and also activates NKT cells via cell wall glycosphingolipids and finally induces cholangitis following exposure in wild-type mice [32]. These results suggest that activated iNKT cells exacerbate PBC-like disease. Herein we demonstrate decreased AMAs, CD4^+^ T, NK, and B cell infiltrates and IFN-γ production of liver mononuclear cells in 2-OA-BSA immunized iNKT cell deficient CD1d ^-/-^ mice.

β-glucosylceramide is a natural plant glycospingolipid and inhibits α-GalCer-mediated activation of NKT cells by binding to its receptor [33]. Administration of β-glucosylceramide ameliorates liver inflammation in TGF- β receptor II dominant-negative (dnTGF- βRII) PBC mice [34]. Of note, administration of either OCH or α-GalCer led to significantly elevated levels of PDC-E2-specific IgM and IgG autoantibodies in 2-OA-BSA immunized mice compared to controls, indicating that activated iNKT cells provide help for antibody production. In addition, 2-OA-BSA immunized CD1d knockout controls have lower levels of AMA and reduced cellular infiltrates compared to controls, suggesting that iNKT cell activation occurs by an endogenous ligand or via the use of complete Freund's adjuvant [35]. Our findings are consistent with our previous studies that activation of iNKT cells by glycolipid antigens enhance autoantibody production. In addition, the lack of iNKT cells will reduce autoantibody production [36,37]. Hence our thesis that iNKT cells regulate autoimmune responses at more than one level.

Studies using models of experimental autoimmune diseases such as arthritis, diabetes, and experimental autoimmune encephalomyelitis (EAE) have indicated that activation of iNKT cells by OCH ameliorates or prevents these Th1-mediated diseases, attributed to induction of IL-4 and Th2 skewing [9,10,11,12]. However, in this study, we found OCH exacerbates the manifestations of autoimmune cholangitis in 2-OA-BSA immunized mice to approximately the same levels observed with administration of α-GalCer. The pathogenesis of organ-specific autoimmune diseases has been previously thought to be orchestrated by Th1 and/or Th17, not Th2 cells [38]. PBC is considered a Th1 and/or Th17 dominant autoimmune responses. In the serum of patients with PBC, the most significant increases were noted for IFN-γ and IL-17, although increased levels of IL-2, IL-4, IL-5, and IL-10 have also been reported [16,39,40,41,42,43]. In addition, an increased in the frequency of IL-17^+^ lymphocytic infiltration in liver has also been noted [40,42]. Our results suggest that activation of other immune networks by activated NKT cells may be equally important for the pathogenesis of cholangitis. Thus, the importance of Th subsets and cytokines in disease progression requires further study involving IFN-γ, IL-4 and IL-17 and/or blocking of cytokine signals by cytokine-neutralizing antibodies.

In patients with PBC, there are increased number of liver NK cells [44]. We report herein that NK cells are increased in both α-GalCer and OCH injected 2-OA-BSA immunized mice while decreased in CD1d^-/-^ mice immunized with 2-OA-BSA. In a previous study, administration of polyI:C, a viral RNA mimetic and Toll-like receptor 3 agonist, to activate NK cells in 2-OA-BSA immunized mice, induces profound exacerbation of cholangitis [45]. In fact, long term administration of polyI:C alone also induces a PBC-like disease [46]. Moreover, NK cells isolated from PBC patients have greater ability to kill autologous biliary epithelial cells than those from patients with other liver diseases [47], suggesting that NK cells may be involved in bile duct damage of PBC. However, depletion of NK cells and NKT cells by intravenous injection of NK1.1 Abs in 2-OA-BSA immunized PBC mice, demonstrate that there is a marked suppression of AMAs and cytokine production of T cells without change in portal inflammation [48]. Taken together, our data reinforce the concept that iNKT cells has a critical influence in the pathogenesis of autoimmune cholangitis.
